# Clinical Trials Update in Resectable Esophageal Cancer

**DOI:** 10.3390/cancers18020300

**Published:** 2026-01-19

**Authors:** Aaron J. Dinerman, Shamus R. Carr

**Affiliations:** 1 Department of Surgery, Baylor University Medical Center, Dallas, TX 75246, USA; 2 Thoracic Surgery Branch, National Cancer Institute, National Institutes of Health, Bethesda, MD 20892, USA

**Keywords:** esophagus, adenocarcinoma, squamous cell carcinoma, induction therapy, adjuvant therapy, chemotherapy, immunotherapy, radiation therapy, esophagectomy, robot

## Abstract

Management of resectable esophageal cancer has evolved into a multidisciplinary paradigm centered on multimodality therapy. Historically, induction chemoradiotherapy followed by surgery, as established by the CROSS trial, became the standard of care for locally advanced disease due to improvements in R0 resection rates and overall survival. More recently, the ESOPEC trial reexamined this paradigm in esophageal adenocarcinoma, demonstrating superior survival and improved systemic disease control with perioperative chemotherapy using the FLOT regimen compared with chemoradiotherapy. In parallel, the MATTERHORN trial further advanced perioperative treatment by showing improved event-free survival with the addition of the immune checkpoint inhibitor durvalumab to FLOT chemotherapy. Alongside these systemic therapy advances, surgical management has transitioned toward minimally invasive and robotic-assisted esophagectomy, offering equivalent oncologic outcomes with reduced perioperative morbidity. This review summarizes the evolving evidence from pivotal clinical trials, highlights ongoing studies integrating immunotherapy, and discusses emerging strategies such as adoptive cell transfer which currently is under investigation for metastatic recurrence, but in the future may provide additional treatment options for resectable esophageal cancer.

## Simple Summary

The treatment of resectable esophageal cancer has undergone substantial transformation over the past decade, driven by advances in systemic therapy, immunotherapy, and surgical technique. Multimodal treatment remains the cornerstone of care for locally advanced disease, traditionally combining induction chemotherapy or chemoradiotherapy followed by esophagectomy. More recently, immune checkpoint inhibitors have emerged as a major therapeutic advance, being incorporated into both induction and adjuvant treatment strategies. These agents have demonstrated meaningful improvements in pathologic response rates, disease-free survival, and event-free survival in selected patient populations. At the same time, surgical management has evolved with the widespread adoption of minimally invasive and robotic-assisted esophagectomy, offering equivalent oncologic outcomes with improved perioperative recovery and reduced morbidity. This review summarizes the current evidence supporting modern multimodality treatment approaches for resectable esophageal cancer, highlights key completed and ongoing clinical trials, and explores emerging strategies such as adoptive cell transfer therapy that may further refine personalized treatment in the future.

## 1. Introduction

Cancer of the esophagus has been known since ancient times; however, it was not until 1913 that Dr. Franz Torek was the first to successfully utilize surgery in the treatment of esophageal cancer. Since then, surgical innovations have progressed [1] from pioneers such as Zaaijer, to Oshawa, to Belsey, to Lewis, to McKeown, to Orringer, and to Luketich. Despite these innovators, long-term results for patients undergoing resection alone for esophageal cancer remained poor. It was advancements in chemotherapy in the 1970s that began to demonstrate improvements in survival.

One of the first trials to explore neoadjuvant chemotherapy was the American INT 113 trial [2]. This trial randomized esophageal cancer patients to chemotherapy either before or after surgery. While there was no significant difference in 5 year survival between the two groups, the small subset of patients that received the chemotherapy prior to surgery, who had a pathologically complete response (pCR), achieved significantly longer survival. The MRC-EOE2 randomized trial [3] reported results in 2002 and demonstrated an absolute improvement in overall survival (OS) of 9% at 2 years in patients receiving preoperative chemotherapy compared to the control group. These results persisted [4] when at a median follow-up of six years a continued significant improvement was seen in the experimental arm with a hazard ratio of 0.84.

Initial studies which included radiation therapy in the neoadjuvant setting suffered from increased presurgical mortality or postoperative morbidity. Eventually trials began to report improvements in patient perioperative outcomes and slight increases in OS. With accumulating results from trials, a meta-analysis by Gebski et al. [5] confirmed both safety of this regimen and acceptable surgical outcomes. Then in 2012 the eagerly awaited results of the CROSS trial [6] were published. This demonstrated that preoperative chemoradiotherapy improved survival compared to surgery alone in patients with resectable esophageal cancer. These pivotal results remained the standard until 2024 when the CROSS trial was compared to induction chemotherapy alone (ESOPEC trial), but only in esophageal adenocarcinoma [7].

The dawn of immunotherapy and combining it with chemotherapy or chemoradiotherapy, in either the induction or adjuvant setting, has begun to emerge as the likely next advance in the management of esophageal cancer. This article will review the current results and ongoing trials for the treatment of esophageal cancer while placing them in the context of the increased use of minimally invasive surgery.

## 2. Induction Therapy

After over 20 years of various randomized trials evaluating induction chemoradiotherapy, most trials did not demonstrate a survival benefit. However, an even greater number of debates occurred critical of trial designs, sample sizes, and poor surgical outcomes. A meta-analysis by Gebski et al. [5] of eight randomized controlled trials of neoadjuvant therapies confirmed both the safety of neoadjuvant chemotherapy and chemoradiotherapy, but also that preoperative chemoradiation followed by surgery could reduce the relative risk of death by 19% and doubled two-year survival rates compared to MRC-EOE2 trial. This resulted in the preferred paradigm becoming induction chemoradiotherapy followed by esophagectomy for resectable patients [6], compared to either induction chemotherapy followed by surgery or surgery followed by adjuvant therapies. This trimodal approach became the standard of care for the past 13 years for esophageal cancer, which has helped to improve 5 year survival from 19% to nearly 50% over the past 30 years [8]. In 2024 with the initial report of the ESOPEC trial [7], and subsequent publication in 2025 of the MATTERHORN trial [9], the paradigm has again shifted.

### 2.1. What Chemotherapy Combination to Use in the Induction Setting?

Early trials utilized various combinations of chemotherapeutic drugs. Finally, in 2015 a systematic review [10] concluded that paclitaxel plus a platinum-based drug was more efficacious than a platinum-based drug combined with 5-fluorouracil, especially in squamous cell carcinoma (ESCC) patients. More recently, the FLOT4 trial [11] evaluated Fluorouracil plus Leucovorin, Oxaliplatin, and doceTaxel against either epirubicin and capecitabine combined with either oxaliplatin or 5-fluorouracil (ECF/ECX) in resectable gastric and gastroesophageal junction adenocarcinoma (this study did not include upper or mid esophageal tumors). The 716 patients with resectable disease were equally divided. The median OS was 50 months vs. 35 months, favoring the FLOT regimen. FLOT is an intensive regimen that may be de-escalated to dual therapy with a platinum agent plus a taxane. Additional trials since 2019 have confirmed FLOT as first-line chemotherapy for esophageal cancer [7,9,12]. In addition, the understanding of microsatellite instability (MSI) and its role in treatment choice in esophageal cancer is evolving [13]. It appears that patients that are MSI-high may benefit more from immunotherapy [14].

### 2.2. Induction Therapy with Chemotherapy Combined with Surgery

At about the same time, trials were underway that were evaluating induction chemoradiotherapy; other trials were evaluating induction chemotherapy with surgery to surgery alone. Results from the various trials were mixed. The RTOG 8911 trial [15] did not show a difference in patients undergoing induction chemotherapy followed by surgery compared to surgery alone, which may have been because nearly 40% of patients did not receive an R0 resection. However, patients with objective tumor response pathologically after induction chemotherapy did have improved survival. The key take home message from this trial was the value in achieving an R0 resection.

Shortly thereafter, a randomized trial of ESCC from Japan [16] reported a 60% 5 year survival compared to 38% favoring those that received induction compared to adjuvant chemotherapy, respectively. Additional trials [17,18] continued to report the benefits of induction chemotherapy followed by surgery in the most common histological subtypes of esophageal cancer. Boonstra et al. [17] focused on ESCC and reported a median OS of 16 months with induction chemotherapy compared to 12 months in the surgery alone group and 5 year OS of 26% and 17%, respectively. In a trial that focused on adenocarcinoma of the esophagus, Ychou et al. [18] reported 5 year OS of 38% vs. 24%, again favoring induction chemotherapy followed by surgery to surgery alone.

The summary of these trials was that induction chemotherapy can increase the R0 resection rate and it is likely that this increase alone plays the greatest contribution to improvements in OS seen with induction chemotherapy compared to surgery alone. However, these trials also highlight the complexity of esophageal cancer trials with different chemotherapy regimens, different histological makeup, and that adenocarcinoma is more prevalent in the West while ESCC is more prevalent in Asia.

The publication of the ESOPEC trial answered two important questions. First, it reinforced induction chemotherapy with FLOT as first-line therapy. Second, by randomizing surgical patients to either induction FLOT or the CROSS trial protocol, it provided data to evaluate the role of radiation therapy as part of an induction therapy regimen. This phase III randomized trial demonstrated significantly improved both overall and progression-free survival for esophageal adenocarcinoma patients who received induction chemotherapy alone compared to the CROSS regimen. At 3 years, the FLOT arm achieved an OS of 61.1% compared to 55.9% and, more notably, a reduced rate of distant recurrence (31.5% vs. 47.2%). The findings indicate that FLOT offers better systemic disease control and long-term outcomes, supporting its use as a more effective perioperative strategy in this patient population [7].

### 2.3. Induction Chemoradiotherapy Combined with Surgery

The trial that conclusively demonstrated the value of induction therapy and became the treatment paradigm for over a decade was the CROSS trial [6], which compared induction chemoradiotherapy (CRT) followed by surgery to surgery alone in patients with potentially resectable esophageal cancer. One of the key differences in this trial was the use of 41.4 Gy of radiation, compared to 50.4 Gy, which had previously been used. The preliminary results demonstrated that the R0 rate increased significantly to 92%, compared to 69% in the surgery alone arm. Additionally, 29% of patients had a complete pathologic response. Complications were similar in both groups. The median survival in the induction CRT group was 49.4 months, compared to 24.0 months in the surgical group. A few years later, mature results of the CROSS trial [8] reported similar median survival overall, but the ESCC group that underwent CRT had a median survival of 81.6 months, compared to 21.1 months for surgery alone. The adenocarcinoma group was also statistically significant, but with median survival of 43.2 months vs. 27.1 months, for induction CRT followed by surgery to surgery alone. This confirmed both the value of induction CRT followed by surgery and that the benefit was conferred to both adenocarcinoma and squamous subtypes. However, with the publication of the ESOPEC trial, which only evaluated esophageal adenocarcinoma, the CROSS trial remains the standard at present for ESCC. However, this paradigm may once again be changed by immunotherapy in the near future.

## 3. Immunotherapy

Due to significant advances in immune checkpoint blockade, the natural progression in the treatment of esophageal cancer is now to look at trials that utilize this type of immunotherapy either in the induction setting or as part of adjuvant therapy after surgery. This is, in part, due to the nearly 70% of patients who do not have a pCR and eventually develop recurrence. If given as part of induction therapy, it may increase the pCR rate, or if in the adjuvant setting, it may decrease recurrence rates. Previous clinical trials [19,20] of advanced gastroesophageal cancers not amenable to resection that were previously treated demonstrate improved survival in patients receiving immunotherapy compared to either placebo or chemotherapy alone. Publication of the MATTERHORN trial in July 2025 demonstrated adding the checkpoint inhibitor durvalumab to the induction therapy FLOT regimen in resectable esophageal adenocarcinoma further improves event-free survival compared to placebo [9].

### 3.1. Immunotherapy in the Induction Setting

At present, there are few robust clinical trials that utilize immunotherapy in the induction setting in patients with operable disease. However, by first looking at the results of using immunotherapy upfront in patients with unresectable disease, such as in the Keynote-590 trial, we see improvements in OS and progression-free survival (PFS) in all patients and particularly in those with squamous cell histology. The recently published results of the MATTERHORN trial identified a meaningful improvement in event-free survival (EFS) when durvalumab was added to FLOT, compared to placebo, in resectable esophageal adenocarcinoma [9]. The median EFS was not reached in the arm with durvalumab and was 32.8 months in the placebo plus FLOT arm. At 24 months, the EFS was 67.4% compared to 58.5% when receiving durvalumab. Additionally, the pCR was 19.2% with the addition of durvalumab compared to 7.2% without (*p*-value < 0.001).

There are a few other trials exploring the role of immunotherapy in the induction setting with mixed results. Notably, the results of EA2174 were reported in 2024 at the American Society of Clinical Oncology meeting and failed to demonstrate an improvement in pCR rate with the addition of nivolumab to induction chemoradiotherapy followed by surgery. The Keynote-585 trial for esophageal, esophagogastric, and gastric adenocarcinoma was also reported in 2024 [12]. While only about a fifth of the enrolled patients had esophageal cancer, the addition of pembrolizumab to induction FLOT also improved pCR rates but failed to demonstrate a meaningful improvement in EFS.

As it specifically relates to ESCC, there is a phase 1 trial (PALACE-1) that evaluated induction CRT with pembrolizumab [21]. This trial reported a meaningful pCR after surgery. However, another completed trial (ACTS-29), but without published results and with the same regimen, shows a mixed picture. The success of PALACE-1 prompted the investigators to open PALACE-2 [22]. Additionally, a phase 3 trial (AIRES) is recruiting in China that randomizes patients after esophagectomy to adjuvant chemotherapy plus an immune checkpoint inhibitor or to adjuvant monotherapy with the immune checkpoint inhibitor [23]. These and future trials (Table 1) may help to define the role of immune checkpoint inhibitors in resectable ESCC.

While the future looks bright for the inclusion of immunotherapy into likely both the induction and adjuvant settings in the treatment of resectable esophageal cancer, there remain challenges. Trials will have to work to establish the optimal treatment strategy and determine if immunotherapy should be given alone or in combination with chemotherapy and/or radiation. Then, should immunotherapy be given only as part of induction or adjuvant, or should it be used before and after surgery? Also, after surgery, should it be given to all patients or only those that do not achieve a pathological complete response? Another issue is that early results appear to indicate a difference in response for squamous cells and adenocarcinoma histologies to immune checkpoint inhibitors. It will be important to investigate biomarkers to accurately aid in the clinical evaluation of patients receiving these drugs. Finally, as more advances are made, there will undoubtably be more drugs, and sorting out which ones are most efficacious to try and tailor the therapy to the individual patient will need to be explored.

### 3.2. Induction Chemoradiotherapy Followed by Surgery Combined with Adjuvant Immunotherapy

While utilizing checkpoint inhibitor immunotherapy as part of an induction regimen has yet to report results, there are results as part of adjuvant treatment after induction chemoradiotherapy and surgery in esophageal cancer patients [24]. CheckMate-577 was a global, randomized, double-blinded, placebo-controlled trial of patients who had undergone induction CRT followed by R0 surgical resection and had residual disease pathologically. These patients were randomized to either nivolumab, a PD-1 checkpoint inhibitor, or placebo for a year, with the primary end point being disease-free survival. At a median follow-up of 24.4 months, patients who received adjuvant nivolumab had a median disease-free survival of 22.4 months compared to 11.0 months for those patients who received placebo. The beneficial effect was more pronounced in those with ESCC as opposed to gastroesophageal tumors, and with complete response at the tumor despite residual disease in a lymph node. Importantly, nivolumab was well tolerated although side effects requiring need to discontinue the trial were slightly higher in the experimental arm (9% vs. 3%). It should also be noted that there was clear benefit in patients regardless of PD-L1 expression, with minimal difference between patient subgroups stratified by PD-L1 expression [24].

These early and encouraging results have shown there will likely be a role for immunotherapy in the multimodal treatment approach to esophageal cancer. Recent publications and ongoing trials will further shape this landscape.

## 4. Vaccine Trials

Vaccines are generally designed to prevent future disease by prophylactically introducing weakened or non-viable vectors with preserved immunogenicity to induce a durable adaptive immune response. Therapeutic vaccines can be utilized in cancer to treat existing malignancies by developing the vaccine directly from cancer cell antigens. While the concept is laudable, results have generally been lacking. This is likely due to multiple factors, including finding optimal antigens and tumor-mediated immunosuppression.

Recently, therapeutic vaccines for esophageal cancer have focused on two well-known cancer-testis antigens [25,26]: New York Esophageal Squamous cell carcinoma 1(NY-ESO-1) and Melanoma-Associated antigen A (MAGE-A). Both of these cancer-testis antigens have demonstrated overexpression in esophageal cancer patients [27,28,29]. While early phase studies [30,31,32] have shown that therapeutic vaccines for esophageal cancer are well tolerated and can have promising anti-tumor activity, results of survival benefits are lacking.

The concept of combining cancer antigen vaccines with immunotherapy [33] has shown some promise with those in the combination treatment group, demonstrating a better antibody response compared to vaccine alone. There is an ongoing phase I/II trial at the National Institutes of Health in Bethesda, Maryland looking at an esophageal cancer lysate vaccine combined with Entinostat and Nivolumab (NCT05898828).

## 5. Adoptive Cell Transfer (ACT)

Adoptive cell transfer (ACT) is an adaptive immune strategy that has the potential to create a cancer-specific, enhanced, and sometimes uniquely personalized treatment. ACT can utilize Tumor-Infiltrating Lymphocytes (TIL), T-Cell Receptor-engineered T-cells (TCR-T), or Chimeric Antigen Receptor T-cells (CAR-T). All three of these final cellular products are ultimately infused into the patient, usually in an inpatient monitored setting and sometimes in combination with other immunomodulatory drugs (including cytokines and/or checkpoint inhibitors). The first reports of TIL, TCR-T, and CAR-T were in 1988, 2006, and 2011, respectively [34,35,36]. Since then, great strides have been made utilizing this strategy and has expanded to many other malignancies [37,38,39] with some striking results. While CAR-T therapy has seen its greatest successes in hematologic cancers such as leukemia and lymphoma [40], TCR-T has had success in metastatic melanoma and sarcoma while TIL therapies have had a profound impact on the treatment of metastatic melanoma [41]. While there are no results, there currently are ongoing clinical trials of ACT that include unresectable esophageal cancer or patients who develop metastatic recurrence.

### 5.1. Tumor-Infiltrating Lymphocytes (TIL)

**Figure 1:**
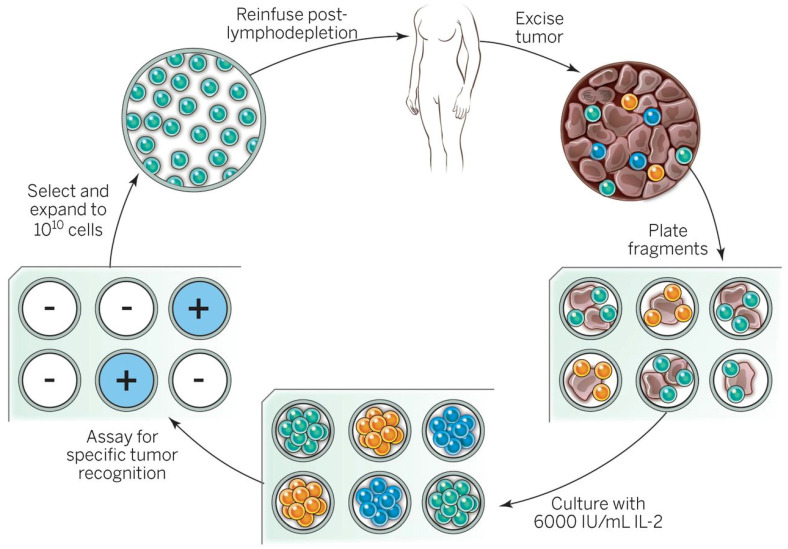
General schema for using the adoptive cell transfer of naturally occurring autologous TILs.

TIL, the original adoptive cellular therapy first pioneered by the surgeon Dr. Steve A. Rosenberg in the 1970s, involves ex vivo culture of T-cells derived from surgically harvested patient tumors with subsequent infusion into the patient. TIL has evolved since its initial development to include an intensive manufacturing process to create a product with potent anti-tumor activity. Specific testing of TIL “fragments” for anti-cancer activity may be one strategy to improve response rates for various metastatic solid epithelial cancers, including breast, cholangiocarcinoma, and colorectal adenocarcinoma [42,43,44]. Anecdotal reports have demonstrated durable regressions using selected TIL for these diseases. TIL is not typically a genetically engineered cell therapy product, although there are active trials accruing patients with metastatic solid epithelial cancer that involve genetic engineering of ex vivo cultured TIL (NCT044266690). The theoretical advantages of TIL are the natural TCR-based effector immunity of anti-cancer adaptive immunity (TCR-T’s advantage over CAR-T) combined with the ability to create a cell product composed of a heterogeneous population of anti-cancer T-cells that potentially target multiple cancer antigens (Figure 1). A variety of accruing clinical protocols evaluating TIL permit inclusion of metastatic esophageal cancer; however, to our knowledge TIL has yet to be tested in the perioperative setting for any resectable solid tumor histology.

The resected melanoma specimen is digested into a single-cell suspension or divided into multiple tumor fragments that are individually grown in IL-2. Lymphocytes overgrow, destroy tumors within 2 to 3 weeks, and generate pure cultures of lymphocytes that can be tested for reactivity in coculture assays. Individual cultures are then rapidly expanded in the presence of excess irradiated feeder lymphocytes, OKT3, and IL-2. By approximately 5 to 6 weeks after resecting the tumor, up to 1011 lymphocytes can be obtained for infusion into patients. (Used with permission without edits from Rosenberg et al. [41]. Permission from Copyright Clearance Center, Inc.)

### 5.2. T-Cell Receptor-Engineered T-Cells (TCR-T)

**Figure 2:**
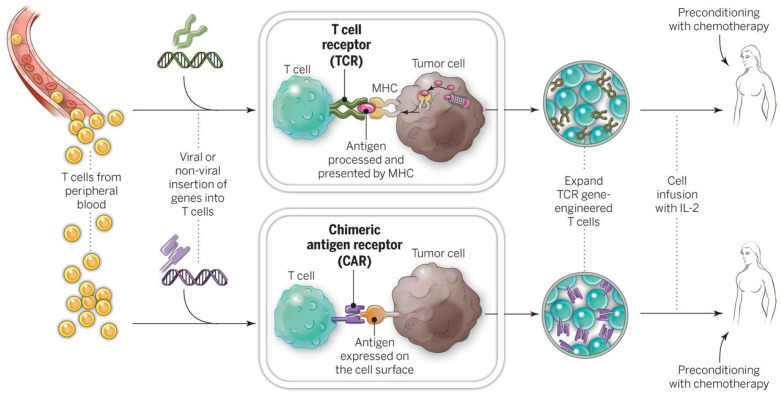
Gene-modification of peripheral blood lymphocytes.

TCR-T employs genetically modified T-cells that are engineered to express a fully functional TCR that is specific to cancer antigens that are presented in the context of HLA. In contrast to CAR-T, using TCR-T theoretically allows for the recognition of any cancer protein product that can be presented by the TCR’s matching HLA/MHC receptor. In contrast, CAR-T is restricted to targeting proteins naturally expressed on the cell surface (Figure 2). This broadens the antigens capable of being recognized to any overexpressed or mutated cancer protein, these do not need to be expressed on the surface of the cancer (as in CAR-T). While many TCRs are often initially discovered naturally in index patients [39,44], their genetic sequence can be altered for affinity enhancement to hypothetically improve in vivo effectiveness [45]. Most TCR-T is based on MHC/HLA Class 1-restricted receptors that are based in CD8 T-cells. This is based on two classic tenets of basic immunology: the hypothetical favorability of the directly cytotoxic CD8 T-cell vs. the indirect helper CD4 T-cell and the intrinsic ability of cancer cells to naturally express Class I, but not Class II, MHC/HLA.

The evolution of TCR-T targeting of neoantigens, unique somatic mutations creating epitopes not shared by healthy tissue, has evolved out of earlier toxicity with TCR-T against shared antigen such as CEA [37,38,39,46,47]. Trials continue in multiple centers using TCR-T, for which esophageal cancer patients may be eligible. KRAS is an attractive target for TCR, as it is a driver oncogene expressed in many cancers; however, various KRAS mutations occur only in up to 17% of esophageal cancers [48] and seem to vary by histology, with it more common in adenocarcinoma subtypes [49]. Different KRAS-specific TCR-T trials are currently recruiting for metastatic solid epithelial cancer patients (including esophageal). If successful, this may provide a role for TCR in the adjuvant setting in high-risk patients that are likely to recur after surgery despite induction therapy.

To broaden the reach of ACT to other cancers, techniques are being developed to introduce antitumor receptors into normal T-cells that could be used for therapy. The top panel shows the insertion of a conventional TCR into a patient’s T lymphocytes, followed by the expansion and infusion back into the patient. The bottom panel shows the insertion of a CAR into a patient’s T-cell, followed by the expansion of these cells and their re-infusion. TCRs and CARs are fundamentally different in their structures and in the structures that they recognize. TCRs are composed of one a chain and one b chain, and they recognize antigens that have been processed and presented by one of the patient’s own MHC molecules. CARs are artificial receptors that can be constructed by linking the variable regions of the antibody heavy and light chains to intracellular signaling chains (such as CD3-zeta, CD28, 41BB) alone or in combination with other signaling moieties. CARs recognize antigens that do not need to be MHC-restricted, but they must be presented on the tumor cell surface. (Used with permission without edits from Rosenberg et al. [41]. Permission from Copyright Clearance Center, Inc.)

### 5.3. Chimeric Antigen Receptor T-Cells (CAR-T)

Perhaps the most recognized form of ACT is CAR-T therapy, although it is the most recently developed iteration of adoptive cellular immunotherapy. CAR-T involves collection of peripheral blood lymphocytes from patients and genetically engineers their expression of a chimeric antigen receptor (CAR). A CAR is composed of the external recognition structure of an antibody (antigen-binding site) fused with the internal effector structure of a T-Cell Receptor (TCR). These chimeric antigen receptors then recognize and bind to specific proteins on the surface of the cancer to induce T-cell-mediated cytotoxicity. Unfortunately, CAR-T success in solid tumors, as in esophageal cancer, has been disappointing. The reasons for this include a paucity of targetable surface antigens unique to solid tumors, cross-reactivity of shared antigens with normal healthy tissue causing severe immune-related adverse events (on-target, off-tumor toxicity), and the uniquely immunosuppressive tumor microenvironment of some solid epithelial malignancies. In other words, most antigens on the surface of solid tumors can also be found on the surface of some vital non-tumor tissue, which would preclude the use of CAR-T. Cross-reactivity has been demonstrated in CAR-T targeting cancer markers such as HER-2 (ERBB2) [50]. Despite this, there are phase I and II trials evaluating the role of CAR-T in solid tumors that can include, or are specific for, unresectable esophageal cancer: NCT03706326, NCT03740256, NCT03013712, NCT04581473.

## 6. Surgical Trials

Initial comparisons of various open techniques were confined to either meta-analysis [51], large database analysis [52], or a few randomized trials [53,54,55,56,57,58]. Despite various flaws in each, the consistent conclusion was that there is no oncologic difference between transhiatal and transthoracic esophagectomy.

While the first report of a minimally invasive approach to esophagectomy [59] was described as far back as 1995, it was the innovations and advances of Dr. James Luketich and the group at the University of Pittsburgh that finally helped to usher in the wide-spread adoption of minimally invasive esophagectomy (MIE). Dr. Luketich was able to demonstrate that in over 1000 patients MIE offered a 30 day mortality of 1.7%, and 0.9% in the second 500 patients, a very low conversion to open and enviable length of stay at the time [60]. These results were corroborated by a multi-institutional study (ECOG 2202) [61]. Innovation has continued its march forward as surgeons have adopted the use of the robotic platform from Intuitive Surgical^TM^ for esophageal resections as the next frontier in minimally invasive esophageal surgery.

By definition, a MIE is an approach that is totally laparoscopic in the abdomen and thoracoscopic in the chest, while a robotic-assisted minimally invasive esophagectomy (RAMIE) has the chest and abdominal portions performed on a robotic platform. There are descriptions of both transhiatal and transthoracic minimally invasive approaches, but this is beyond the scope of the review. However, when comparing these very small series, the results are similar to the conclusions of the comparisons of the two open techniques.

Since initial reports of MIE, the percentage of esophagectomies currently performed in a minimally invasive fashion has been increasing [62]. While there are some regional variations in the United States, over half are performed in this fashion. During this same period, numerous retrospective reports comparing RAMIE to either open or MIE techniques have been published. Because of the inherent limitations of retrospective comparative studies, the focus will be on prospective trials or propensity matched analyses. Although, a recent systematic review of this topic with a meta-analysis [63] does sum up all the studies. Lastly, while surgeon familiarity, training, and hospital resources are factors in determining which minimally invasive technique an individual surgeon might offer a patient, there are some differences.

### 6.1. RAMIE vs. Open Esophagectomy

First, looking at RAMIE vs. various open techniques [64,65,66,67,68], while some were early feasibility studies that reported short-term outcomes [65,67], others reported either 3 or 5 year OS and DFS (Table 2). In the only prospective randomized trial with long-term results [64], at a median follow-up of 60 months, long-term oncologic outcomes were equivalent for recurrence rate, OS, and patterns of recurrence, which is consistent in the propensity match trials. However, it is some specific short-term outcomes that demonstrate the advantages of RAMIE when comparing to open techniques in terms of length of stay, blood loss, improved lymph node dissection, complications (especially pulmonary), and improved quality of life. The issue of reported vocal cord palsy differs widely between the trials, but this appears to come down to the definition used to define how severe the palsy must be to be reported as a complication.

### 6.2. RAMIE vs. MIE

There are several observational studies [69,70,71,72,73,74,75,76] that compare various aspects and outcomes of RAMIE and MIE and then utilize propensity matching to further compare the two surgical techniques (Table 3). One study [77] performs a retrospective review of a prospective database comparing the two minimally invasive approaches, and there is a single randomized controlled trial that only evaluated ESCC [78]. In addition, there are a few studies [79,80,81] of large databases that compare RAMIE, MIE, and open techniques. However, none of the studies are randomized controlled trials.

While the number of patients and the mix of adenocarcinoma and squamous cell carcinoma differ between these reports, the overall findings from these studies essentially report similar short-term outcomes for estimated blood loss, R0 resection rates, overall complications, 90 day mortality, operative time, and length of stay. Studies with long-term follow-up report that long-term oncologic outcomes are also similar. However, results regarding lymph node harvest and vocal cord palsy between RAMIE and MIE differ. All studies demonstrate less vocal cord palsy issues with RAMIE compared to MIE. What is interesting is that this incidence for RAMIE is improved when compared to earlier studies comparing it to open esophagectomy. The cause of this is unknown but may be due to surgeon learning curve or increased precision with the robotic platform. Several of these studies report an increased number of lymph nodes being removed with RAMIE vs. MIE. This is backed up in a meta-analysis [83] that favors RAMIE for an increased lymph node harvest. However, since recommendations are that at least 15 lymph nodes be obtained during esophagectomy, both MIE and RAMIE exceed this value. Additionally, the increased number of lymph nodes resected by RAMIE does not equate to meaningful improvements in location of recurrence, DFS, or OS.

In the few studies that include both open techniques and minimally invasive techniques, the results continue to demonstrate the same findings; minimally invasive techniques, regardless of RAMIE or MIE, are associated with better short-term outcomes and lower complications compared to open techniques, and that there is little difference to separate MIE and RAMIE outcomes. Therefore, at present, while minimally invasive approaches to esophagectomy should be offered compared to open, the choice between MIE and RAMIE rests with the experience of the surgeon and the resources of the hospital.

## 7. Conclusions

The multimodal treatment paradigm of esophageal cancer is currently evolving. It appears that for resectable esophageal adenocarcinoma, induction chemotherapy with the immune checkpoint inhibitor durvalumab is the current paradigm. However, for ESCC induction, chemoradiotherapy without immunotherapy followed by surgery should still be the first-choice regimen. Immunotherapy in esophageal cancer has demonstrated in various clinical trials that it is effective and well tolerated by patients and provides meaningful improvements in survival. The results of the CheckMate-577 and MATTERHORN are likely just the beginning in the paradigm shift that is occurring in the treatment of esophageal cancer. Future trials will help to define whether immunotherapy should be given prior to surgery or as an adjuvant, or possibly both. There is also the possibility that immunotherapy may supplant the role of radiation in the induction setting, as has occurred with non-small cell lung cancer. The role of ACT in the resectable setting is unexplored, but some anecdotal reports in the metastatic setting offer an intriguing avenue for research.

From a surgical standpoint, the clear benefits of minimally invasive surgery no longer require debate. However, there are a few issues from a surgical standpoint that need to be addressed. First, it will be important to see how induction immunotherapy impacts surgical outcomes and complications. Another issue will be if radiation continues to have a role in the induction setting. Then, the optimal time to perform surgery after completing induction therapy will need to be ascertained. Finally, if pathological complete response rates significantly increase, this may alter utilizing surgery to only those patients requiring salvage esophagectomy.

## Figures and Tables

**Table 1:** Trials of ESCC utilizing immune checkpoint inhibitors combined with surgery.

Clinical Trial	Study Design	Regimen	Enrollment	Results or Outcome Measures
PALACE-1 [21] (NCT03792347)	Phase 1—single arm	Induction CRT + pembrolizumab	N = 20	pCR 55.6%
PALACE-2 * [22] (NCT04435197)	Phase 1/2—single arm	Induction CRT + pembrolizumab with postoperative pembrolizumab	N = 143	pCR is primary endpoint. Disease-free survival and 3 year OS are secondary endpoints
ACTS-29 ** (NCT02844075)	Phase 2—single arm	Induction CRT + pembrolizumab with postoperative pembrolizumab	N = 28	pCR 23.1%
AIRES * [23] (ChiCRT2100045651)	Phase 3—randomized	Adjuvant chemotherapy + tisleizumab vs. tisleizumab alone	N = 220	Disease-free survival

* Results not yet published; ** results reported on clinicaltrials.gov but not published.

**Table 2:** Studies comparing RAMIE vs. Open Esophagectomy.

Study	Study Design	Evaluated Patients (N)		Induction (%)		Overall Survival (%)		Disease-Free Survival (%)		R0 (%)		Pneumonia (%)		Leak Rate (%)		Vocal Cord Palsy (%)	
		RAMIE	OPEN	RAMIE	OPEN	RAMIE	OPEN	RAMIE	OPEN	RAMIE	OPEN	RAMIE	OPEN	RAMIE	OPEN	RAMIE	OPEN
van Hillegersberg et al. [64,65], 2020 and 2019	Randomized Controlled Trial	54	55	80	75	41 ^◊^	40 ^◊^	43 ^◊^	42 ^◊^	93	96	**28**	**55**	46	40	9.0	11.0
Kwon et al. [66], 2025	Propensity Matching	443	443	28.4	29.3	70.7 ^◊^	55.0 ^◊^	63.3 ^◊^	50.1 ^◊^	NR	NR	2.9	5.6	**2.5**	**5.2**	**22.6**	**14.0**
Sarkaria et al. [67], 2019	Non-randomized prospective	64	106	75	82	NR	NR	NR	NR	96.9	97.2	**14.1**	**34**	3.1	9.4	**3.1**	**0.0**
Yun et al. [68], 2020	Propensity Matching	130	241	**42.3**	**16.2**	81.7 *	73.7 *	49.2 *	45.6 *	97.7	96.7	**3.8**	**10.8**	3.1	2.9	25.4	19.9

* 3 year reported value; ^◊^ 5 year reported value; RA—RAMIE; OE—open esophagectomy; NR—not reported; values in **BOLD** represent reported *p*-values < 0.05 in referenced paper.

**Table 3:** Studies comparing RAMIE vs. MIE.

Study	Study Design	Evaluated Patients		Induction (%)		5 Year Overall Survival (%)		5 Year Disease-Free Survival (%)		Pneumonia (%)		Leak Rate (%)		Vocal Cord Palsy (%)		Total Lymph Nodes Removed	
		RAMIE	MIE	RAMIE	MIE	RAMIE	MIE	RAMIE	MIE	RAMIE	MIE	RAMIE	MIE	RAMIE	MIE	RAMIE	MIE
Chen et al. [69], 2019	Propensity Matching	54	54	25.9	31.5	NR	NR	NR	NR	14.8	24.1	9.3	3.7	**13.0**	**31.5**	NR	NR
Deng et al. [70], 2018	Propensity Matching	79	72	NR	NR	NR	NR	NR	NR	9.6	7.7	5.8	3.8	**13.5**	**7.7**	**20.6**	**17.9**
Ekeke et al. [71], 2023	Propensity Matching	64	181	84.6	80.7	50	40	55	25	17.0	16.6	4.6 ^^^	3.9 ^^^	0.0	1.5	**32** **(25,39)**	**29 (22,26.5)**
Meredith et al. [77], 2020	Retrospective Review of Prospective Database	144	95	77.8	73.4	NR	NR	NR	NR	**6.9**	**23.4**	2.8	5.0	NR	NR	**20 ± 9**	**12 ± 6**
Sakurai et al. [82], 2024	Propensity Matching	26	26	31.8	31.8	73.1	80.8	76.9	76.9	3.8	7.7	15.4	0.0	15.4	38.5	25 (10–57)	28 (5–60)
Tagkalos et al. [74], 2019	Propensity Matching	40	40	86.0	68.0	NR	NR	NR	NR	15.0	17.5	12.5	12.5	NR	NR	27 (13–84)	23 (11–48)
Oshikiri et al. [73], 2021	Propensity Matching	51	51	57	59	NR	NR	NR	NR	NR	NR	14	12	**8**	**21**	22 (11–34)	23 (12–37)
Yang et al. [75], 2020	Propensity Matching	271	271	10.7	10.3	77 *	76 *	77 *	70 *	8.9	12.5	11.8	14.4	**29.2**	**15.1**	20.3 ± 9.9	19.2 ± 9.6
Yang et al. [78], 2022	Randomized Controlled Trial	181	177	21.6	20.9	NR	NR	NR	NR	9.9	11.9	12.2	11.3	32.6	27.1	23 (16–33)	23 (14–30)
Zhang et al. [76], 2019	Propensity Matching	66	66	NR	NR	NR	NR	NR	NR	6.1	7.6	7.6	4.5	6.1	4.5	19.2 ± 9.2	19.3 ± 9.5

* 3 year; ^^^—grade 3 or higher RAMIE—Robotic-Assisted Minimally Invasive Esophagectomy; MIE—Minimally Invasive Esophagectomy (Laparoscopic/Thoracoscopic); NR—not reported; values in **BOLD** represent reported *p*-values < 0.05 in referenced paper.

## Data Availability

No new data were created or analyzed in this study. Data sharing is not applicable to this article.

## Abbreviations

The following abbreviations are used in this manuscript:

ACT	Adoptive Cell Transfer
CAR	Chimeric Antigen Receptor
CAR-T	Chimeric Antigen Receptor T-cells
CEA	Carcinoembryonic Antigen
CRT	Chemoradiotherapy
DFS	Disease-Free Survival
ECF	Epirubicin, Capecitabine, and 5-Flourouracil
ECX	Epirubicin, Capecitabine, and Oxaliplatin
EFS	Event-free survival
ESCC	Esophageal squamous cell carcinoma
FLOT	Fluorouracil plus Leucovorin, Oxaliplatin, and doceTaxel
HLA	Human Leukocyte Antigen
IL-2	Interleukin 2
MAGE-A	Melanoma-Associated antigen A
MHC	Major Histocompatibility Complex
MIE	Minimally Invasive Esophagectomy
MSI	Microsatellite Instability
NR	Not Reported
NY-ESO-1	New York Esophageal Squamous cell carcinoma 1
OKT-3	Muromonab-CD3 (monoclonal antibody)
OS	Overall Survival
pCR	Pathologic Complete Response
PD-1	Programmed Cell Death Protein 1
PD-L1	Programmed Death Ligand 1
RAMIE	Robotic-Assisted Minimally Invasive Esophagectomy
RLN	Recurrent Laryngeal Nerve
TCR	T-cell Receptor
TCR-T	T-cell Receptor engineered T-cells
TIL	Tumor Infiltrating Lymphocytes
